# Hydroxyzine Dihydrochloride Premedication Is a Necessity for Pediatric Patients Undergoing Strabismus Surgery: An Observational Prospective Clinical Trial

**DOI:** 10.1155/2022/4137144

**Published:** 2022-09-21

**Authors:** Fatma Ferda Kartufan, Nurcan Kizilcik, Sule Ziylan, Ferdi Menda

**Affiliations:** ^1^Department of Anesthesia and Reanimation, Hospital of Medistanbul, Istanbul, Turkey; ^2^Department of Anesthesia and Reanimation, Faculty of Medicine, Yeditepe University, Istanbul, Turkey; ^3^Department of Ophthalmology, Faculty of Medicine, Yeditepe University, Istanbul, Turkey; ^4^Department of Anesthesia and Reanimation, Faculty of Medicine, Yeditepe University, Istanbul, Turkey

## Abstract

**Objective:**

In this single-blind, observational prospective clinical trial, we aimed to determine and compare the effects of premedication with hydroxyzine plus midazolam and midazolam alone on the incidence of oculocardiac reflex (OCR).

**Methods:**

Forty-five patients were divided into three groups. Group M received 0.5 mg/kg midazolam alone, Group H received 0.5 mg/kg hydroxyzine plus 0.5 mg/kg midazolam, and Group HM received 1 mg/kg hydroxyzine plus 0.5 mg/kg midazolam. The Ramsay Sedation Scale (RSS), the heart rates (HR1: after induction of anesthesia; HR2: before retraction of orbital muscle; and HR3: right after retraction of orbital muscle), the muscles with OCR, and the incidence of OCR (20% decrease of the HR right after the traction) were recorded and compared between the three groups.

**Results:**

The mean HR1, HR2, and HR3 values were significantly increased (*p*=0.002, *p* < 0.001, *p* < 0.001) and the incidence of OCR (*p*=0.004) was significantly decreased in Group H and in Group HM (for all, *p* < 0.01) compared to Group M. The most common orbital muscle in which OCR occurred was the rectus medialis.

**Conclusion:**

Premedication with a combination of 0.5 or 1 mg hydroxyzine with 0.5 mg midazolam significantly reduced the incidence of OCR compared to premedication with midazolam alone. This study was registered on https://clinicaltrials.gov/ with number NCT03806270.

## 1. Introduction

Strabismus is defined as any deviation of binocular alignment that can be the cause or the effect of poor binocularity [1]. If left untreated timely in children, it may have dramatic consequences on their educational ability, impairing their physical and physiological performance and leading to cosmetic problems [2, 3]. The prevalence of strabismus is reported to be between 2 and 4% of the world's pediatric population [4]. The mainstay of strabismus treatment is surgery. Early strabismus surgery could not only reduce damage to binocular visual function resulting from strabismus but also facilitate the restoration of visual function after surgery [5].

The oculocardiac reflex (OCR) is defined as a decrease in heart rate greater than 20% compared to the baseline following globe pressure or traction of the extraocular muscles [6]. The OCR is a trigeminal-vagal reflex triggered by the stimulation of the ophthalmic branch of the trigeminal nerve during strabismus surgery. Vagal stimulation reduces heart rate and may cause sinus bradycardia, ventricular fibrillation, atrioventricular block, and asystole [7]. The incidence of OCR during strabismus surgery has been reported to be between 56% and 68% [8]. The incidence of OCR decreases with age, and the pediatric population is at the highest risk [6]. Therefore, OCR is one of the serious challenges faced by pediatric anesthesiologists during strabismus surgery.

It has been reported that an increased anesthesia depth influences subcortical areas and prevents the development of OCR through the suppression of nociceptive and autonomic reflexes [9] and that OCR can be prevented by retrobulbar block and with anticholinergic medications [1]. Several studies in the literature have investigated the effects of using various anesthetic agents in pediatric strabismus surgery on the incidence of OCR. According to the results of these studies, propofol or remifentanil anesthesia was associated with a higher incidence of OCR compared to sevoflurane and desflurane when midazolam was used as the induction agent [10]. Dexamethasone administered as nasal prophylaxis increased the rate of OCR [11]; ketamine and midazolam showed similar effects but did not reduce the incidence of OCR [12]. On the contrary, not administering neuromuscular blockers and premedication with benzodiazepine have been reported to reduce the risk of OCR by 3.64 and 3.11 times, respectively [9]. In addition, atropine premedication has been reported to reduce OCR and prevent undesired effects of dysrhythmias [13]. In another study, the incidence of OCR was found to be significantly lower with sevoflurane compared to midazolam [14].

Hydroxyzine dihydrochloride is an antihistamine that crosses the blood-brain barrier and reduces activity in the central nervous system, and thus, it can be used in premedication as a sedative to treat anxiety and tension before surgery. To our best knowledge, there is no study in the literature to evaluate the effect of hydroxyzine dihydrochloride on the incidence of OCR. In this study, we aimed to investigate the effects of premedication with hydroxyzine dihydrochloride plus midazolam on the incidence of OCR in comparison with midazolam alone.

## 2. Materials and Methods

This study was designed as a single-blind, observational, prospective clinical trial and registered with the clinical trials number NCT03806270. Before the beginning, the study protocol was approved by the local ethics committee with 09/09/2018 dated and 884 numbered decision. All participants and their parents were informed in detail about the objectives of the study and gave written informed consent. The study was conducted in accordance with the relevant ethical principles of the Declaration of Helsinki (DoH).

**Figure 1:**
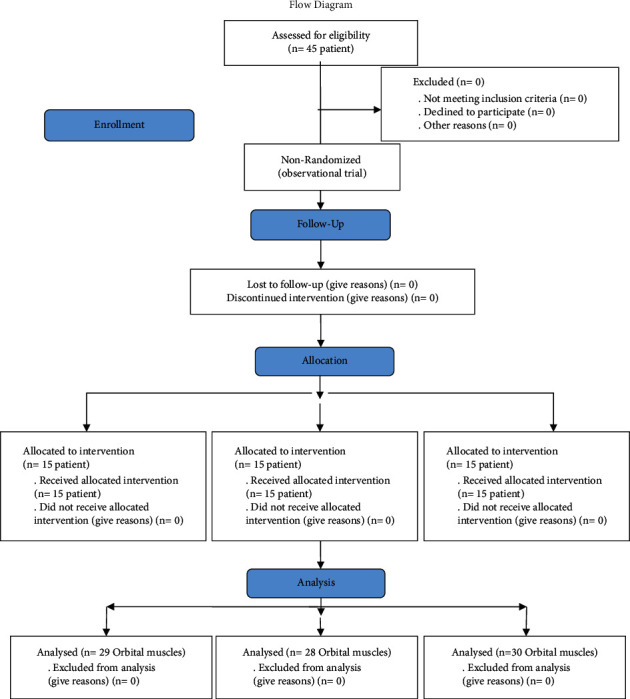
Flowchart of the inclusion.

A total of 75 American Society of Anesthesiologists (ASA) I class patients, aged between 1 and 10 years, and scheduled for strabismus surgery in Yeditepe University Hospital and Yeditepe University Eye Center 2018 and 2019 were included in the study. Patients with a greater ASA status, those who were scheduled for another surgery, patients younger than one year and older than 10 years of age, and those with a history of chronic illness, arrhythmia, or glaucoma (narrow-angle) were excluded from the study. In addition, patients for whom premedication was not ordered or any medication other than those used in the study was ordered for premedication were also excluded. A flowchart of the inclusion is given in Figure 1.

The researcher collecting the raw data was made blind to the study and the patients were grouped. Which sedative was administered to the patients was learned by examining the patient's service file after the study data were obtained. Patients were grouped after study data were obtained. Since the number of patients between the groups was equal, a post hoc test was applied, and when the difference between the groups was found to be statistically significant, the study was terminated because the primary outcome criterion of the study (either reaching the required number or reaching statistical significance between the groups) was reached.

All surgeries were performed by the same surgeon. The most commonly preferred premedications in our anesthesiology clinic for sedation of pediatric patients include 0.5 mg/kg oral midazolam combined with hydroxyzine dihydrochloride in different doses (0.5 mg/kg or 1 mg/kg) or 0.5 mg/kg oral midazolam alone with cherry juice of a maximum 5 mL. In the present study, we aimed to compare the effects of these three combinations on OCR.

Patients administered midazolam alone (0.5 mg with cherry juice) were assigned to Group M, those given 0.5 mg midazolam plus 0.5 mg/kg hydroxyzine dihydrochloride to Group H, and patients who received 0.5 mg midazolam plus 1 mg/kg hydroxyzine dihydrochloride to Group HM.

### 2.1. Study Sampling

Seventy-five pediatric patients were planned to be included in this observational study based on a 20% decrease in OCR compared to the baseline value, using an effect size of 0.40, *α*: 0.05, and power: 0.8 values; the number of patients that should be included in each group was calculated as 25 using G power 3.0.10. However, after reaching 45 patients (15 in each group), the post hoc test was done to evaluate the significance between the oral premedication utilized and the OCR occurrence, HR1, HR2, HR3, and RSS values. Because there was significance in more than one group (between oral premedication, and OCR, oral premedication and HR2, and oral premedication and HR3), the study was completed before reaching the exact number of participants.

### 2.2. Data Collection

The analyzed study data included the Ramsay Sedation Score (RSS) determined preoperatively between 1 point indicating “anxious or restless or both response to stimulus” and 6 points showing “no response to stimulus.” The heart rate-1 (HR1) shows the lowest heart rate observed from electrocardiography (ECG) monitorization at the “time-out” check, after the anesthesia induction and before starting the surgery. The heart rate-2 (HR2) was observed from the ECG monitor when the surgeon warned the researcher just before the traction of each orbital muscle. The heart rate-3 (HR3) was the lowest rate observed from the ECG monitor within 120 seconds after each orbital muscle traction, the OCR occurrence, and the orbital muscle on which traction was performed during surgery.

The anesthesiologist of the patient who ordered oral premedication for sedation before the surgery was not included in the trial. Patients' demographic data and premedications were obtained retrospectively by the researcher from the patients' files after the strabismus surgery. According to the observational setting of the trial, the participants were not randomized and no control group was enrolled.

### 2.3. Statistical Analysis

Data obtained in this study were statistically analyzed using the SPSS version 23.0 (SPSS, Statistical Package for Social Sciences, IBM Inc., Armonk, NY, USA) statistical software. Demographic data of the patients were expressed as mean ± standard deviation and minimum and maximum values. Post hoc examination was carried out with the one-way ANOVA and chi-square tests in order to evaluate the differences between the groups in terms of HR1, HR2, HR3, OCR, and RSS. A comparison of HR1, HR2, and HR3 values between the premedication groups was made with the independent samples *t*-test. The chi-square and Fisher's exact tests were used to determine the significance of OCR incidence between the premedication groups and to evaluate the RSS according to the oral premedication utilized. *P* < 0.05 values were considered statistically significant.

## 3. Results

A total of 45 pediatric patients who underwent strabismus surgery with premedication were included in the study, with 15 patients in each group. Of all the patients, 21 (46.7%) were female and 24 (53.3%) were male. The mean age of the patients was found as 4.2 ± 2.6 years. The mean height was found to be 102.5 ± 18.4 cm and the mean weight was measured to 17.1 ± 8.1 kg. Demographic data of the patients based on gender are given in Table 1.

**Figure 2:**
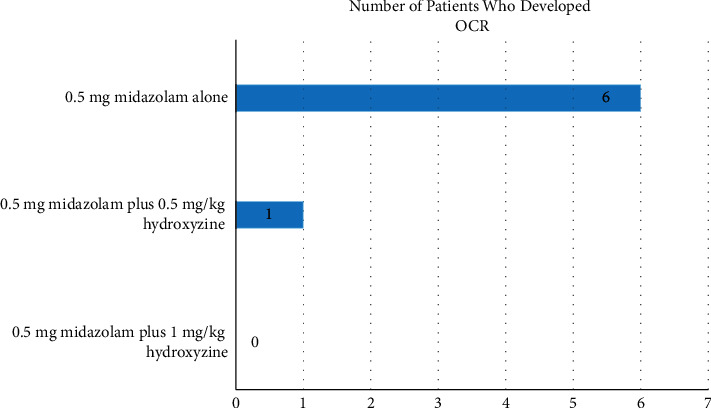
The incidence of OCR between the groups.

The main finding of this study was that administration of premedication with both 0.5 mg midazolam plus 0.5 mg/kg hydroxyzine and 0.5 mg midazolam plus 1 mg/kg hydroxyzine reduced the incidence of OCR compared to 0.5 mg midazolam alone. OCR occurred in 6 (40%) patients in Group M and one (6.6%) patient in Group H, while none of the patients in Group HM developed OCR (Figure 2). Accordingly, the incidence of OCR was statistically significantly lower in Group H (*p*=0.002) and Group HM (*p*=0.001) compared to Group M. No statistically significant difference was found between Group H and Group HM in terms of the occurrence of OCR (*p* > 0.05).

The mean HR1 was statistically significantly higher in Group HM compared to Group M (*p*=0.017). No statistically significant difference was found between Group H and Group M and between Group H and Group HM (both, *p* > 0.05). The comparison of HR2 and HR3 values based on the orbital muscles was statistically significant between Group M (*n*: 29) and Group H (*n*: 28) and between Group M (*n*:29) and Group HM (*n*:30) (both, *p* < 0.001).

**Figure 3:**
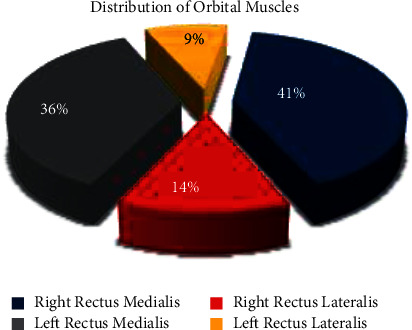
The distribution of orbital muscles in which OCR occurred.

The mean RSS score was statistically significantly higher in Group HM compared to Group H (*p*=0.01). There was no correlation between the RSS scores and the incidence of OCR. The strabismus surgeries were performed on a total of 87 orbital muscles, and OCR occurred in 22 (25.3%) of them. The distribution of the orbital muscles in which OCR occurred is shown in Figure 3. In addition, a comparison of the incidence of OCR related to the orbital muscles according to the premedication groups is shown in Table 2.

## 4. Discussion

The OCR was described for the first time by Aschner in 1908 and is frequently encountered in pediatric strabismus surgery [12]. In the present study, OCR was defined as a 20% decrease in heart rate compared to the baseline value following global pressure. Most OCR resolves without treatment, but it may sometimes cause devastating results such as cardiac arrest and sudden death [15]. The activation of OCR is also associated with noncardiac outcomes such as syncope, hypotensive attacks, emergence agitation, and gastrointestinal reflexes, including postoperative nausea and vomiting (PONV) and emergence agitation [6]. The incidence of PONV following strabismus surgery can rise to 85% in the pediatric population [16].

The manipulation of the extraocular muscles is one of the most important triggers of the development of OCR. The OCR is caused by traction applied to the extraocular muscles, pressure on the globe, ocular trauma, or traction on the conjunctiva. Traction applied especially on the rectus medialis has been associated with the occurrence of OCR [17]. Multiple studies have reported an increased incidence of OCR with medial rectus traction compared to other ocular muscles [18, 19]. Similarly, in our study, the right and left rectus medialis were the most common muscles in which OCR occurred by 77%.

It has been reported that not administering premedication before pediatric strabismus surgery is associated with a 3.11-time increase in the risk of developing OCR [9]. Therefore, it is important to understand and manage OCR with proper premedication for the anesthesiologist. An effective premedication can reduce the incidence of OCR as well as PONV, length of stay, costs, and workload on the healthcare staff.

In the present study, a combination of both 0.5 mg and 1.0 mg hydroxyzine with 0.5 mg midazolam significantly reduced the incidence of OCR (*p*=0.002 and *p*=0.001, respectively). Many different anesthetic agents have been used to help decrease the occurrence of OCR with varying incidences. Several studies have shown that premedication with anticholinergic agents such as atropine or glycopyrrolate decreases the incidence of OCR [11, 20, 21]. However, the use of atropine is controversial because it can produce undesirable dysrhythmias, and if it penetrates into the central nervous system, the central anticholinergic syndrome may be triggered [12]. On the contrary, the results reported by different studies on pediatric strabismus surgery are diverse in daily practice [22]. Misurya et al. demonstrated that patients receiving premedication with atropine and retrobulbar block with 2% xylocaine did not develop OCR [23]. There are studies stating that ketamine is effective in reducing the incidence of OCR compared to other anesthetic drugs. Hahnenkamp et al. reported that a combination of ketamine with a volatile anesthetic may be useful for preventing OCR. However, high doses of ketamine are needed in order to achieve this preventing effect [24]. Oh et al. compared the incidence of OCR between ketamine and midazolam. In that study, although the occurrence of OCR was more frequent in the midazolam group, the difference was not statistically significant [12]. Also, the side effects of ketamine, such as hypersalivation and disturbing dreams, are common. For this reason, ketamine is not the first choice in premedication [25]. In another study, Abdelaziz et al. compared intranasal dexmedetomidine or intranasal midazolam and found that midazolam was associated with a higher incidence of OCR, although the difference did not reach statistical significance [26]. Anticholinergic drugs can cross the blood-brain barrier and cause undesirable conditions, such as postoperative delirium, urinary dysfunction, skin rash, fever, and anticholinergic syndrome [27]. Besides, systemic applications of anticholinergic drugs also cause ectopic beats and ventricular arrhythmias, and for this reason, they are not recommended for routine prophylaxis in pediatric ophthalmic surgeries [28].

In the present study, we investigated the effect of 0.5 mg or 1 mg hydroxyzine combined with oral midazolam and midazolam alone on the incidence of OCR for the first time in the literature and found a lower incidence of OCR with both doses of hydroxyzine.

Hydroxyzine acts by suppressing the activity of some subcortical structures, such as the reticular formation and the limbic system. In this way, it relieves the feeling of anxiety and reduces the emotional and somatic response to stress. Its relaxing effect on skeletal muscles, bronchodilator activity, antihistaminic, and analgesic effects are other important positive effects [29, 30]. This work was previously published as a preprint [31].

It has been shown in various clinical studies that hydroxyzine also has antiemetic effects. It also strongly inhibits cutaneous and systemic anaphylaxis. Hydroxyzine is completely metabolized in the liver after rapid absorption from the gastrointestinal tract and is mainly excreted fecally. In this respect, it seems suitable for oral premedication. There is no interaction with atropine, which is also an important advantage for the possibility of using atropine in OCR treatment. When evaluated in these aspects, it may be thought that it may be superior to anticholinergics as a premedication.

### 4.1. Study Limitations

The main limitation of this study is relatively the small number of patients. In addition, several other factors such as the incidence of PONV and length of stay in hospitals could be studied. However, being the first in the literature to investigate the effect of premedication with hydroxyzine on the incidence of OCR in pediatric strabismus surgery makes this study strong and guiding for further studies to be conducted in the future.

## 5. Conclusion

Premedication with a combination of 0.5 or 1 mg hydroxyzine with 0.5 mg midazolam did not only sedate agitated pediatric patients before strabismus surgery but also protected them by reducing the incidence of OCR. However, further comprehensive studies with a larger series of patients are needed to draw firm conclusions.

## Figures and Tables

**Table 1:** Demographic data of the patients based on gender.

	Female (*n* = 21)	Male (*n* = 24)	Total (*n* = 45)
	Mean ± standard deviation		
Age (year)	4.3 ± 2.4	4.2 ± 2.8	4.2 ± 2.6
Height (cm)	102.6 ± 16.1	102.5 ± 20.5	102.5 ± 18.4
Weight (kg)	17.6 ± 7.6	16.7 ± 8.7	17.1 ± 8.1

**Table 2:** Distribution of the orbital muscles with OCR according to the groups.

Premedication group	Orbital muscles with OCR (*n* = 22)	Orbital muscles operated (*n* = 87)	Orbital muscles with OCR/orbital muscles operated	Orbital muscles with OCR/total muscles operated (*n* = 87)
Group M	15 (68.2%)	29	15 (51.72%)	15 (17.24%)
Group H	6 (27.3%)	28	6 (21.4%)	6 (6.90%)
Group HM	1 (4.5%)	30	1 (3.3%)	1 (1.15%)
Total	22 (100%)	87	22 (100%)	22 (25.3%)

## Data Availability

The data used to support the findings of this study are included within the article.

## References

[1] Garcia C. A. D. A., Sousa A. B. D., Mendonça M. B. D. M., Andrade L. L. D., Oréfice F. (2004). Prevalence of strabismus among students in Natal/RN-Brazil. *Arquivos Brasileiros de Oftalmologia*.

[2] Chia A., Dirani M., Chan Y. H. (2010). Prevalence of amblyopia and strabismus in young Singaporean Chinese children. *Investigative Ophthalmology & Visual Science*.

[3] Jackson S., Harrad R. A., Morris M., Rumsey N. (2006). The psychosocial benefits of corrective surgery for adults with strabismus. *British Journal of Ophthalmology*.

[4] Agaje B. G., Delelegne D., Abera E. (2020). Strabismus prevalence and associated factors among pediatric patients in southern Ethiopia: a cross-sectional study. *Journal of International Medical Research*.

[5] Wan X., Wan L., Jiang M., Ding Y., Wang Y., Zhang J. (2021). A retrospective survey of strabismus surgery in a tertiary eye center in northern China, 2014-2019. *BMC Ophthalmology*.

[6] Dunville L. M., Sood G., Kramer J. (2020). Oculocardiac Reflex. *StatPearls*.

[7] Donlon J. V., Doyle D. J., Feldman M. A., Miller R. D. (2006). Anesthesia for eye, ear, nose and throat surgery. *Miller’s Anesthesia*.

[8] Neils D. M., Singanallur P. S., Vasilakis M., Wang H., Tsung A. J., Klopfenstein J. D. (2014). Incidence and ramifications of the oculocardiac reflex during the orbitozygomatic approach: a prospective assessment. *World Neurosurgery*.

[9] Aydın B. G., Küçükosman G., Pişkin Ö (2021). Factors affecting oculocardiac reflex incidence in pediatric strabismus surgery: retrospective study. *JARSS*.

[10] Choi S. R., Park S. W., Lee J. H., Lee S. C., Chung C. J. (2009). Effect of different anesthetic agents on oculocardiac reflex in pediatric strabismus surgery. *Journal of Anesthesia*.

[11] Arnold R. W., Jansen S., Seelig J. C., Glasionov M., Biggs R. E., Beerle B. (2021). Anesthetic impacts on the oculocardiac reflex: evidence from a large, observational study. *Clinical Ophthalmology*.

[12] Oh A., Yun M., Kim H., Kim H. (2007). Comparison of desflurane with sevoflurane for the incidence of oculocardiac reflex in children undergoing strabismus surgery. *British Journal of Anaesthesia*.

[13] Gilani S. M., Jamil M., Akbar F., Jehangir R. (2005). Anticholinergic premedication for prevention of oculocardiac reflex during squint surgery. *Journal of Ayub Medical College, Abbottabad*.

[14] Çeliker V., Dal D., Saricaoglu F., Başgül E., Aypar Ü (2004). Comparison of the effects of midazolam and sevoflurane on oculocardiac reflex. *Türk Anesteziyoloji ve Reanimasyon Dernegi Dergisi*.

[15] Ha S. G., Huh J., Lee B. R., Kim S. H. (2018). Surgical factors affecting oculocardiac reflex during strabismus surgery. *BMC Ophthalmology*.

[16] Watcha M. F., Simeon R. M., White P. F., Stevens J. L. (1991). Effect of propofol on the incidence of postoperative vomiting after strabismus surgery in pediatric outpatients. *Anesthesiology*.

[17] Deriy L., Gerstein N. S., Panikkath P., Ram H., Starr B. (2019). Cardiac patients requiring emergent noncardiac surgery. *Essentials of Cardiac Anesthesia for Noncardiac Surgery*.

[18] Shakil H., Wang A. P., Horth D. A., Nair S. S., Reddy K. K. V. (2019). Trigeminocardiac reflex: case report and literature review of intraoperative asystole in response to manipulation of the temporalis muscle. *World Neurosurgery*.

[19] Başağaoğlu B., Steinberg A., Tung I. T., Olorunnipa S., Maricevich R. S. (2018). Oculocardiac reflex as a late presentation of orbital floor fracture. *Journal of Craniofacial Surgery*.

[20] Rahimi Varposhti M., Moradi Farsani D., Ghadimi K., Asadi M. (2019). Reduction of oculocardiac reflex with Tetracaine eye drop in strabismus surgery. *Strabismus*.

[21] Ducloyer J. B., Couret C., Magne C. (2019). Prospective evaluation of anesthetic protocols during pediatric ophthalmic surgery. *European Journal of Ophthalmology*.

[22] Gayer S., Tutiven J. (2006). Anesthesia for pediatric ocular surgery. *Ophthalmol Clin North Am*.

[23] Misurya V. K., Singh S. P., Kulshrestha V. K. (1990). Prevention of oculocardiac reflex (O.C.R) during extraocular muscle surgery. *Indian Journal of Ophthalmology*.

[24] Hahnenkamp K., Hã–nemann C. W., Fischer L. G., Durieux M. E., Muehlendyck H., Braun U. (2000). Effect of different anaesthetic regimes on the oculocardiac reflex during paediatric strabismus surgery. *Pediatric Anesthesia*.

[25] White P. F., Way W. L., Trevor A. J. (1982). Ketamine its pharmacology and therapeutic uses. *Anesthesiology*.

[26] Abdelaziz H. M. M., Bakr R. H., Kasem A. A. (2016). Effect of intranasal dexmedetomidine or intranasal midazolam on prevention of emergence agitation in pediatric strabismus surgery: a randomized controlled study. *Egyptian Journal of Anaesthesia*.

[27] Joo J., Lee S., Lee Y. (2014). Emergence delirium is related to the invasiveness of strabismus surgery in preschool-age children. *Journal of International Medical Research*.

[28] Rodgers A., Cox R. G. (2010). Anesthetic management for pediatric strabismus surgery: continuing professional development. *Canadian Journal of Anesthesia/Journal canadien d’anesthésie*.

[29] Guaiana G., Barbui C., Cipriani A. (2010). Hydroxyzine for generalised anxiety disorder. *Cochrane Database of Systematic Reviews*.

[30] (2012). *Bethesda (MD) LiverTox: Clinical and Research Information on Drug-Induced Liver Injury*.

[31] https://www.researchsquare.com/article/rs-1165795/v1.

